# Antibody Libraries as Tools to Discover Functional Antibodies and Receptor Pleiotropism

**DOI:** 10.3390/ijms22084123

**Published:** 2021-04-16

**Authors:** Chih-Wei Lin, Richard A. Lerner

**Affiliations:** Department of Chemistry, The Scripps Research Institute, La Jolla, CA 92037, USA; cwlin@scripps.edu

**Keywords:** combinatorial antibody library, agonist antibody, cell fate, phage display

## Abstract

Most antibodies currently in use have been selected based on their binding affinity. However, nowadays, antibodies that can not only bind but can also alter the function of cell surface signaling components are increasingly sought after as therapeutic drugs. Therefore, the identification of such functional antibodies from a large antibody library is the subject of intensive research. New methods applied to combinatorial antibody libraries now allow the isolation of functional antibodies in the cellular environment. These selected agonist antibodies have provided new insights into important issues of signal transduction. Notably, when certain antibodies bind to a given receptor, the cell fate induced by them may be the same or different from that induced by natural agonists. In addition, combined with phenotypic screening, this platform allows us to discover unexpected experimental results and explore various phenomena in cell biology, such as those associated with stem cells and cancer cells.

## 1. Introduction

Antibodies, also known as immunoglobulins (Ig), are mainly produced by B cells, and can specifically target antigens. After Köhler and Milstein developed hybridoma technology in 1975, the speed of antibody development in both basic and clinical research accelerated markedly [1]. Since the first antibody (muromonab CD3, Orthoclone OKT3) was approved by the Food and Drug Administration (FDA) in 1986, antibodies have become an important category of biopharmaceutical products. To date, more than 80 antibodies have been approved for clinical use in the treatment of various human diseases, including many cancers, autoimmune, metabolic, and infectious diseases [2,3].

Currently, most therapeutic antibodies have been discovered, selected, and developed from animal immunization, B cell cloning, or the use of combinatorial antibody libraries. One of the reasons for the success of combinatorial antibody libraries is that they utilize the characteristics of the natural immune system. Every individual, even before antigen stimulation, carries the genetic information for and the capacity to produce more than 10^7^ different antibodies. Moreover, despite an initial low affinity, the antigen binding sites of many of these antibodies can cross-react with a variety of related, but different antigens. The subsequent affinity maturation resulting from repeated stimulation in the continued presence of an antigen leads to higher affinity antibodies capable of binding to the “new” antigen. Thus, an initial naïve repertoire can be expanded many-fold.

**Figure 1:**
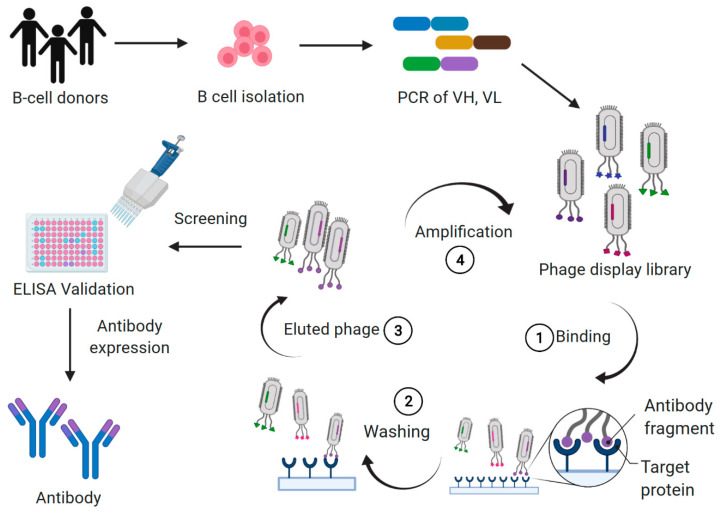
The illustration of phage combinatorial antibody libraries. Lymphocyte cells are collected from humans, e.g., naïve, cancer patients, disease survivors carrying antibodies with unique characteristics, or immunized animals. The RNA of B cells is prepared and transcribed into single-stranded cDNA that is used as the source for PCR amplification of the heavy chain (VH) and the light chain (VL) genes. Variable genes are cloned into phagemid vectors as antibody fragments, then produced to phage combinatorial antibody libraries in *E. coli* bacteria. The target protein is immobilized on immunotubes for selection. Specific binding phages displaying antibodies are enriched over several selection rounds by 1. binding, 2. washing, 3. elution, and 4. phage amplification. After 3–5 rounds of biopanning, the specific phage binders are screened by ELISA, then DNA sequences are analyzed and cloned into appropriate expression vectors to produce antibodies, or various antibody formats for functional analysis.

Antibody phage display is one aspect of combinatorial antibody library technology, and is the in vitro selection technique most commonly used to select antibodies with high affinity for specific antigens. In 1985, George P. Smith described phage display technology, showing that filamentous bacteriophages can display peptides of interest on their surface after peptide-encoding DNA fragments had been inserted into the bacteriophage coat protein genes. Subsequently, many laboratories developed methods for generating antigen-specific antibodies by creating combinatorial antibody libraries in filamentous phages [4,5,6,7,8,9,10]. In general, combinatorial antibody libraries are constructed from mRNA or RNA extracted from B cells of immunized or naïve donors. Because the variable regions of the heavy chain (VH) and the light chain (VL) representing the immunoglobulin gene-encoding repertoire are known, we can use polymerase chain reaction (PCR), and then specific primers to amplify different VH and VL gene families. These variable regions of antibody repertoires are then ligated to the phage display vector (phagemid), with the net result that the antibody is expressed on the surface of the phage and the gene encoding of the antibody is in the phage genome. The antibody-encoding gene is inserted into a phage coat protein gene causing the antibody fragment to be displayed on the outside of the phage, which results in a connection between genotype and phenotype. Once produced, the antibody library in the phage can be bound to an immobilized antigen. This allows us to use multiple binding and elution cycles for selection of antibodies with high affinity and specificity. Phage binders are screened by ELISA, then DNA sequences are analyzed and cloned into appropriate expression vectors to produce antibodies, or various antibody formats, for functional analysis (Figure 1). The antibody format in the phage display library can be a single-chain variable fragment (scFv) or Fab fragments. Due to the small size and high solubility of phage particles (10^13^ particles/mL), a library size of up to 10^11^ independent clones can be effectively generated, and an extensive antibody diversity can be displayed in a single library. One advantage of combinatorial antibody library technology is the diversity of binders. Another advantage is the ability to directly generate human antibodies in vitro. Since many important therapeutic antibodies are antibodies to self-antigens, which is forbidden by tolerance, in vitro generation allows the by-passing of tolerance and the generation of antibodies to self-antigens such as cell surface proteins, or self-products such as Tumor necrosis factor alpha (TNF-α).

Currently there are 11 approved antibodies (Table 1) derived from these combinatorial antibody library technologies [11,12,13,14,15,16,17,18,19,20,21,22,23,24]. The majority of these antibodies are originally generated by few companies, Cambridge Antibody Technology (CAT), Dyax and MorphoSys’s human combinatorial antibody libraries (HuCAL). The most dominant antibody format of the approved or under clinical investigations phage-derived antibodies is Immunoglobulin G (IgG). The therapeutic antibodies from phage libraries can be successfully screened and isolated to treat cancer and non-cancer medical conditions such as inflammatory, infectious, or immune diseases [25]. Nowadays, combinatorial antibody libraries can be very large, often containing more than 10^11^ members. The sheer number and diversity of antibodies in such libraries increases the likelihood that searches to select binders specific for any one of a wide range of antigens will be successful. In brief, the process usually involves multiple rounds of affinity selection against the desired target, followed by antibody purification, binding analysis, and functional testing. Many antibodies derived from combinatorial libraries that simply bind antigens have been shown to be extremely important. For example, Adalimumab (an antibody that binds to tumor necrosis factor) is the world’s best-selling drug [26].

Most antibody libraries are now made in the form of human single-chain variable fragments (scFv) or Fab for the isolation of therapeutic antibodies. Some of the antibody formats, i.e., the single domain antibody fragments (also referred to VHH, sdAb, Nanobodies), have also developed [27,28,29,30,31,32,33]. These formats can also be used to find functional antibodies in cell fate, cellular pathways, viral pathways, or G protein-coupled receptor (GPCR) structure. In addition, in respect of the intracellular expression of antibodies in cells, for example, Visintin et al. used the two-hybrid [28] and Cattaneo et al. [34] used intrabody in vivo systems to provide an understanding of individual antigen–antibody fragments that function in cells. The phenotypic screening of nanobody libraries was also used for isolated antiviral VHH that protect human A549 cells from lethal infection with influenza A virus (IAV) or vesicular stomatitis virus (VSV). This study helps us to understand the viral life pathways [35]. These studies have shown that antibody fragments can efficiently interact with their antigens in vivo. Thanks to the development of new technology, today’s researchers can use these 10^11^ binding antibodies in extracellular, intracellular, or cell membrane systems. Antibodies can be secreted, expressed in the cytoplasm, anchored on the plasma membrane, or implanted in the endoplasmic reticulum [36,37,38]. Using such delivery technology, we can expand the applicability of combinatorial antibody libraries. Like the phage display system, we continue to emphasize the link between genotype and phenotype. However, this time the link is in an animal cell. When the combinatorial antibody library is used to infect eukaryotic cells, the integrated antibody genotype and cell phenotype are permanently connected, and each cell becomes its own selection system. Therefore, we can manipulate antibody libraries to study fundamental biological processes in the cell environment.

Over the years, most antibodies have been screened for molecular targets. Cell-based phenotypic analysis is another new method that can be used to explore biological relevance. This review will highlight recent discoveries using human scFv combinatorial antibody libraries in target-based screening for agonist antibodies, as well as exploring how phenotypic screening can be used to discover functional antibodies that perturb targets involved in cell fate.

## 2. Using Antibody Libraries to Discover Functional Antibodies and Receptor Pleiotropism

### 2.1. Target-Based Screening for Agonist Antibodies

**Figure 2:**
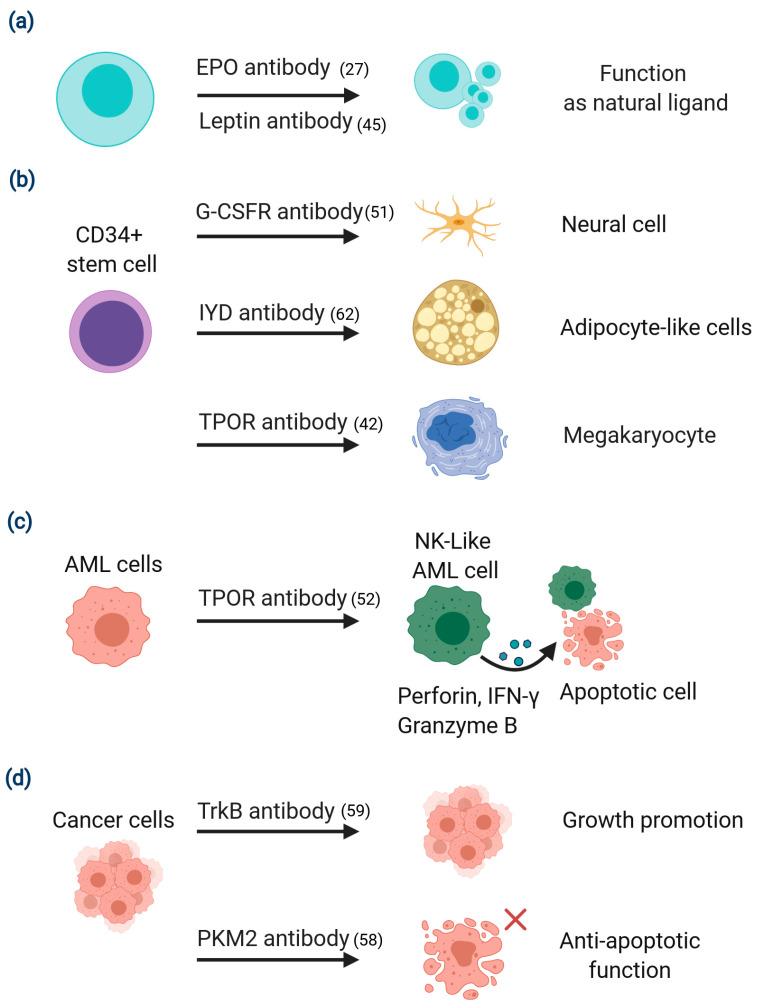
Some examples of antibody libraries as a tool to discover functional antibodies and receptor pleiotropism. Functional antibodies can isolate from target-based or phenotypic-based screening from a combinatorial antibody library. Agonist antibodies that are generated against signaling receptors often induce the same cellular response as the natural agonist ligand for the receptor, or the agonist antibody induces a different cell fate than the natural ligand, even though they both bind to the same receptor. (**a**) The erythropoietin (EPO) agonist or the leptin agonist antibody have the same biological activity as a natural ligand. (**b**) Exposure of CD34+ bone marrow stem cells with different functional agonist antibodies, e.g., Granulocyte Colony Stimulating Factor Receptor (G-CSFR) specific agonist antibody, iodotyrosine deiodinase (IYD) antibody, and thrombopoietin receptor (TPOR) antibody can differentiate into nerve cells, adipocyte-like cells, and megakaryocytes, respectively. (**c**) Exposure of acute myeloid leukemia (AML) cells to TPOR antibody promotes their differentiation into natural killer (NK)-like AML cells that can induce apoptosis in non-differentiated AML cells. (**d**) Exposure of cancer cells to TrkB antibody or PKM2 antibody that promote tumor growth or anti-apoptotic function. Abbreviations: erythropoietin (EPO), Granulocyte Colony Stimulating Factor Receptor (G-CSFR) thrombopoietin receptor (TPOR), acute myeloid leukemia (AML), natural killer (NK), interferon-γ (IFN-γ), iodotyrosine deiodinase (IYD), tropomyosin receptor kinase B (TrkB), pyruvate kinase M2 (PKM2).

In general, most agonist antibodies are obtained by a target-based approach, i.e., by focusing on a specific cellular signal by targeting its receptor. An antibody is then selected that mimics the natural ligand or modulates the effect of the targeted receptor. Previously the initial step in antibody screening was solely based on binding, e.g., hybridomas. The subsequent production, isolation, and identification of clones to obtain functional antibodies was a very laborious and time-consuming process. Now, experimenters are able to directly select antibodies on the basis of their function or mechanism of action, e.g., antibodies with enzyme-like catalytic activity [39] or antibodies with agonist activities capable of binding to desired targets and activating downstream signaling [40,41]. Agonist antibodies directed against cell surface targets have become one of the most effective ways to mimic natural ligands or enhance immune responses, because they can also lead to antibody receptor-mediated downstream signaling in cells. For this purpose, a combinatorial antibody library can be used to screen autoantigens. We can transfer antibody genes to a lentivirus system, with all antibody genes constructed with a membrane anchor sequence, and then use them to infect target animal cells. The integrated antibody genotype and cell phenotype will be permanently connected, and each cell will be available for further selection via fluorescence-activated cell sorting (FACS). If we use known targets, pre-selecting antibodies can improve antibody production efficiency. In 2012, Zhang et al. described a method to screen for erythropoietin (EPO) receptor (EPOR) antibodies from a library of combinatorial antibodies that bind to the EPO receptor with similar activity to the natural ligand, EPO [27]. Based on this concept, many function-based antibodies with high potency and full agonist activity have been developed recently [42,43,44,45,46], and their use in the clinical setting has a number of advantages over present practice. For example, there are several challenges that accompany long-term clinical use of growth factors or cytokines. These include short serum half-life, low bioavailability, dose-limiting toxicity and immunogenicity. An example of an agonist antibody overcoming these issues is the selection of an antibody targeting the leptin receptor, which was shown to be of high potency with full agonist activity and a function similar to leptin (Figure 2a). In this work the leptin antibody was first selectively enriched by phage display and later screened and isolated using leptin receptor reporter cells. This antibody showed identical biochemical properties and cellular profiles as leptin, and rescued leptin-deficiency in ob/ob mice [45].

Recently, antibodies targeting inhibitory immune checkpoints have been shown to be very effective in cancer immunotherapy. Furthermore, evidence suggests that antibodies targeting stimulatory checkpoints, e.g., OX40, 4-1BB, CD27, and ICOS, may be equally successful in cancer therapy [40]. Many agonist antibodies have been derived from hybridomas, however, they have also been selected from antibody libraries. In one example, the researchers first used a phage library and activated human lymphocytes to generate a large collection of antibodies against 10 immune checkpoints, LAG-3, PD-L1, PD-1, TIM3, BTLA, TIGIT, OX40, 4-1BB, CD27, and ICOS. Through next-generation sequencing and bioinformatics analysis, they identified individual scFvs in each collection and then selected the most enriched antibodies. The antibodies were then further confirmed by assays for lymphocyte proliferation and T-cell function [47].

### 2.2. Phenotypic Screening for Isolation of Functional Antibodies That Regulate Cell Fate

A large proportion of drugs in clinical use have been developed by first identifying molecules that have the desired effect on the function of a cell, and then subsequently identifying their targets [48]. However, when the goal is to develop antibodies capable of regulating cell phenotype other methods have been used. In these methods the stem cell is the starting point. Stem cells are highly specialized cells with unlimited replication and self-renewal potential. These cells are pluripotent, but may be limited or unlimited (embryonic stem cells, ESC) in terms of their ability to differentiate into a range of somatic cell lineages. Therapies capable of controlling differentiation have long been a goal in the pharmaceutical industry. They would have broad applicability in the fields of tissue regeneration and the treatment of chronic degenerative diseases [49,50], and would be a major advance in the clinical setting. In one study researchers screened an antibody library to find functional antibodies capable of activating specific receptors on bone marrow stem cells. They successfully selected agonist antibodies recognizing the granulocyte colony stimulating receptor (G-CSFR) by anchoring the expressed antibodies to the membrane of a G-CSFR-transfected BaF3 cell line. Importantly, subsequent experiments showed that one of the isolated anti-G-CSFR antibodies was able to induce CD34+ hematopoietic stem cells to differentiate into nerve cells (Figure 2b) [51]. In other experiments, an agonist antibody that functions like a natural ligand specific for the thrombopoietin receptor (TPOR) stimulated bone marrow stem cells to differentiate into megakaryocytes [42]. Interestingly, exposure of acute myeloid leukemia (AML) cells to this same TPOR agonist antibody promoted their differentiation into natural killer (NK)-like AML cells that synthesized large amounts of perforin, granzyme B and interferon-γ, and thereby induced apoptosis in undifferentiated AML (Figure 2c) [52]. This antibody was shown to induce signal transducer and activator of transcription 3 (STAT-3), protein kinase B (AKT), and extracellular signal-regulated kinase (ERK) phosphorylation in CD34+ hematopoietic stem cells. Similarly, in AML cells, the TPOR antibody induced the same signaling in STAT-3 and ERK phosphorylation, but not in AKT. These findings suggest that the ability to use receptor pleiotropism to change the differentiation state of stem cells may open a new route to treating disease [53]. Melidoni et al. reported the selection of antagonist antibodies capable of blocking fibroblast growth factor 4 (FGF4) and its receptor FGFR1β, which control embryonic stem cell differentiation [54]. In addition, antibodies have been shown to be capable of reprogramming differentiated cells into induced pluripotent stem cells (iPSCs), a process that is usually generated by transient expression of Oct4, Sox2, Klf4, and c-Myc in the nucleus. Using the autocrine antibody reprogramming system from an antibody library, multiple antibodies that replaced either Sox2 and c-Myc in combination, or Oct4 alone in generating iPSCs were isolated. Identifying the target of one Sox2 replacement antibody showed that it binds to Basp1, thereby de-repressing nuclear factors WT1, Esrrb, and Lin28a (Lin28) independently of Sox2. This study provides another example whereby an antibody library can be used as a tool for discovery of new biologics, as well as elucidating membrane-to-nucleus signaling pathways that regulate pluripotency and cell fate [55].

As mentioned above, functional antibodies such as agonists can mimic the actions of natural ligands, having the ability to stimulate proliferation and differentiation. However, occasionally these antibodies, in spite of binding the same target, appear to activate a signaling pathway different from the natural ligand, with the consequence that differentiation occurs along a different cell lineage. This indicates a receptor pleiotropism that is like a binary switch, which can regulate a variety of biological activities. Phenotypic screening offers additional opportunities to identify targets and select antibodies that regulate cell fate or affect tumor growth, and combining phenotypic screening with antibody libraries enhances the chance for success. For example, one of the most important phenotypes in biology is cell death. Using a combination of phenotypic screening and combinatorial antibody libraries, researchers were able to select antibodies that protected cell death associated with rhinovirus infection. The target antigen was later identified as the rhinovirus 3C protease [56]. Mammalian cells exposed to disturbances in the intracellular or extracellular microenvironment can activate one of many signal transduction cascades, ultimately leading to their death. Each of these regulated cell death modes is triggered and spread through a different molecular mechanism, all of which exhibit a considerable degree of complexity [57]. In the cancer setting, a current study reported that one anti-apoptotic intrabody was selected from an antibody combinatorial library, and shown to recognize pyruvate kinase M2 (PKM2). This finding helped to identify a new mechanism that allows cells to evade apoptosis [58] (Figure 2d). Two other important phenotypes in biology are cell proliferation and metastasis. In a recent study, two antibodies derived from a combinatorial library, both recognizing tropomyosin receptor kinase B (TrkB) and highly similar in sequence, were shown to have opposite functional activity, one being an agonist while the other was an antagonist. The agonist antibody was shown to increase breast cancer cell growth both in vitro and in vivo, whereas the antagonist antibody inhibited growth. Receptor binding by the agonist antibody triggered the same downstream signaling cascade as the natural ligand, brain-derived neurotrophic factor (BDNF) [59]. This unexpected finding in TrkB represents yet another example showing that the same receptor may have different functions. A platform for phenotypic discovery of antibodies and targets applied on chronic lymphocytic leukemia (CLL) has been reported [60]. The platform utilizes primary patient cells throughout the discovery process and includes methods for differential phage display cell panning, cell-based specificity screening, phenotypic in vitro screening, target deconvolution, and confirmatory in vivo screening. This approach provides another method of discovering potent targets for antibody-based cytotoxicity treatment of CLL, e.g., CD32, CD21, etc.

These antibodies also show promise for the treatment of degenerative diseases. Recent reports have shown that it is possible to select antibodies from a combinatorial library that can induce bone marrow stem cells to differentiate into microglia, which then traffic to the brain where they organize into typical networks. Interestingly, in an Alzheimer’s disease mouse model, these induced microglia-like cells were found at sites of plaque formation and significantly reduced their plaque numbers [61].

As we mentioned above, these findings suggest that the use of phenotypic screening with combinatorial antibody libraries shows great promise in terms of allowing identification of receptor pleiotropism, as well as selection of antibodies capable of modulating the differentiation, growth, and function of cells [61,62,63]. The ultimate goal is to use this new capability to expand treatment options for both cancer patients and patients with degenerative diseases.

## 3. Antibody Libraries and Emerging Viral Infections

One important advantage of combinatorial antibody libraries is the almost limitless diversity of binding antibodies they contain, potentially greater than that seen in nature. Work focusing on the outbreak of the novel coronavirus disease in 2019 (COVID-19) has shown that this characteristic can be extremely valuable in the development of therapies for emerging viruses [37,64,65,66]. COVID-19 is the biggest global health threat in many generations. Recently it has been reported that a combinatorial human antibody library, constructed 20 years before the current COVID-19 pandemic, was used to select three highly potent antibodies that specifically bind the severe acute respiratory syndrome coronavirus 2 (SARS-CoV-2) spike protein, and neutralize authentic SARS-CoV-2 virus [67]. This suggests that immunological memory after infection with seasonal human coronaviruses may potentially contribute to cross-protection against SARS-CoV-2 [68,69]. Other researchers have successfully isolated neutralizing antibodies from a phage display library constructed using peripheral lymphocytes collected from patients in the acute phase of the disease. These neutralizing antibodies have been shown to recognize different epitopes on the viral spike receptor-binding domain (RBD). Some subset antibodies exert their inhibitory activity by eliminating the binding of RBD to the human angiotensin-converting enzyme 2 (ACE2) receptor. These papers indicated that antibodies from an antibody library represent a promising basis for an effective treatment design for SARS-CoV-2 infection [70,71]. Perhaps most importantly, the large number of antibodies from such libraries allows one to understand the chemistry of virus neutralization.

## 4. Summary

This main purpose of this review was to describe current findings and applications of functional antibodies, especially agonist antibodies, selected from combinatorial antibody libraries. The advent of combinatorial antibody library technology has provided scientists with unprecedented control over the output of the acquired immune system. Antibodies can now be generated in test tubes, thus avoiding tolerance. The size of these libraries, combined with a powerful selection system, allows for rapid generation of antibodies and isolation of rare antibodies. In addition to enhancing traditional uses of antibodies in both research and therapeutics, recent advances have identified functional agonist antibodies capable of activating cell signaling cascades, as well as inducing cell differentiation along multiple pathways, suggesting that in the future antibodies may be used as universal operons for cell function.

## Figures and Tables

**Table 1:** FDA-approved antibody-based drugs from combinational antibody library.

Antibody	Brand Name	Target	Indications	Approved	Company	Ref.
Adalimumab	Humira	TNFα	Rheumatoid arthritis	2002	AbbVie	[11]
Belimumab	Benlysta	BLyS	Systemic lupus erythematosus	2011	GlaxoSmithKline/Human Genome Sciences	[12]
Raxibacumab	Abthrax	B. anthrasis PA	Anthrax infection	2012	GlaxoSmithKline/Human Genome Sciences	[13]
Ramucirumab	Cyramza	VEGFR2	Gastric cancer	2014	Eli Lilly/ImClone Systems	[14]
Necitumumab	Portrazza	EGFR	Non-small cell lung cancer	2015	Eli Lilly/ImClone Systems Inc.	[15,16]
Atezolizumab	Tecentriq	PD-L1	Metastatic lung cancer	2016	Genentech	[17,18]
Avelumab	Bavencio	PD-L1	Merkel cell carcinoma	2017	Merck Serono International S.A./Pfizer	[19]
Guselkumab	Tremfya	IL-23	Plaque psoriasis	2017	MorphoSys/Janssen Biotech Inc.	[20]
Lanadelumab	Takhzyro	PKaI	Hereditary angioedema attacks	2018	Dyax Corp/Shire	[21]
Emapalumab	Gamifant	IFNγ	Primary hemophagocytic lymphohistiocytosis	2018	NovImmmune	[22]
Moxetumomab pasudodox	Lumoxiti	CD22	Hairy cell leukemia	2018	MedImmune/AstraZeneca	[23,24]

Abbreviation: TNF-α, tumor necrosis factor-alpha; BLyS, B-lymphocyte stimulator; B. anthrasis PA, protective antigen of Bacillus anthracis; VEGFR2, vascular endothelial growth factor receptor 2; EGFR, epidermal growth factor receptor; PD-L1, programmed death-ligand 1; IL-23, interleukin-23; pKaI, plasma kallikrein; IFN-γ, interferon gamma.

## Data Availability

The data presented in this study are available on request from the corresponding author.
